# eHealth technologies assisting in identifying potential adverse interactions with complementary and alternative medicine (CAM) or standalone CAM adverse events or side effects: a scoping review

**DOI:** 10.1186/s12906-020-02963-y

**Published:** 2020-07-29

**Authors:** Jeremy Y. Ng, Maryam Mooghali, Vanessa Munford

**Affiliations:** Department of Health Research Methods, Evidence, and Impact, Faculty of Health Sciences, McMaster University, Michael G. DeGroote Centre for Learning and Discovery, Room 2112, 1280 Main Street West, Hamilton, Ontario L8S 4K1 Canada

**Keywords:** Adverse events, Complementary and alternative medicine, eHealth, eHealth technologies, Herb-drug interactions, mHealth, Scoping review, Side effects

## Abstract

**Background:**

While there are several existing eHealth technologies for drug-drug interactions and stand-alone drug adverse effects, it appears that considerably less attention is focussed on that of complementary and alternative medicine (CAM). Despite poor knowledge of their potential interactions and side effects, many patients use CAM. This justifies the need to identify what eHealth technologies are assisting in identifying potential 1) adverse drug interactions with CAM, 2) adverse CAM-CAM interactions or 3) standalone CAM adverse events or side effects.

**Methods:**

A scoping review was conducted to identify eHealth technologies assisting in identifying potential adverse interactions with CAM or standalone CAM adverse events or side effects, following Arksey and O’Malley’s five-stage scoping review framework. MEDLINE, EMBASE, and AMED databases and the Canadian Agency for Drugs and Technologies in Health website were systematically searched. Eligible articles had to have assessed or referenced an eHealth technology assisting in identifying potential one or more of the three aforementioned items. We placed no eligibility restrictions on type of eHealth technology.

**Results:**

Searches identified 3467 items, of which 2763 were unique, and 2674 titles and abstracts were eliminated, leaving 89 full-text articles to be considered. Of those, 48 were not eligible, leaving a total of 41 articles eligible for review. From these 41 articles, 69 unique eHealth technologies meeting our eligibility criteria were identified. Themes which emerged from our analysis included the following: the lack of recent reviews of CAM-related healthcare information; a large number of databases; and the presence of government adverse drug/event surveillance.

**Conclusions:**

The present scoping review is the first, to our knowledge, to provide a descriptive map of the literature and eHealth technologies relating to our research question. We highlight that while an ample number of resources are available to healthcare providers, researchers, and patients, we caution that the quality and update frequency for many of these resources vary widely, and until formally assessed, remain unknown. We identify that a need exists to conduct an updated and systematically-searched review of CAM-related healthcare or research resources, as well as develop guidance documents associated with the development and evaluation of CAM-related eHealth technologies.

## Background

Considerable research has established that the concurrent use of pharmaceutical drugs and complementary and alternative medicines (CAMs) can lead to unwanted interactions; furthermore, certain CAMs have been shown to cause standalone adverse reactions [1]. It is currently estimated that more than 70% of North Americans have tried at least one form of CAM, which includes traditional medicines [2–4], and collectively spend billions of dollars on these therapies each year [5, 6]. An integral part of CAMs include natural health products (NHPs) in Canada, which are sold directly to Canadians and do not require a prescription nor the oversight of a healthcare professional, despite being regulated by Health Canada [7]. A 2010 Ipsos-Reid poll showed that 73% of Canadians took NHPs regularly [8], yet only 19% of Canadians surveyed by Health Canada considered themselves well-informed when purchasing NHPs. Furthermore, 12% of Canadians who used NHPs report that they experienced adverse reactions, and only 41% of Canadians who experienced adverse effects from NHPs reported them [8].

eHealth is used widely in managing patients’ medications, however, it is currently unknown what types of eHealth technologies are available to detect potential drug interactions with CAM or standalone CAM adverse effects. With regard to pharmaceutical technologies, there are some useful medication databases available that can be integrated into hospitals’ electronic data and used as a tool for computerized decision support systems (CDSS). Studies on programs such as the Swedish Finnish Interaction X-referencing or SFINX database have demonstrated a correlation between medication alert systems and a decrease in potentially adverse drug interactions [9]. In some hospitals, medication-related CDSSs are used which evaluate drug dosage, patients’ age, and comorbidities and send alarms when possible drug interactions and side effects are detected [10].

Additionally, there are several mobile apps that provide beneficial information regarding appropriate dosing, potential drug-drug interactions, and side effects. WebMD, Medscape, Epocrates, The Blue Book, and Micromedex are some of the most commonly used mobile drug information apps [11]. These types of mobile health (mHealth) apps are used by both health professionals and patients, and many are publicly available in the Google Play or the Apple App Store [12]. Some mobile apps have been developed to assist patients taking high-risk medications to manage their symptoms. For example, an app was developed by a team of collaborators in Oxford for patients undergoing treatment for colorectal cancer. This type of mHealth system shares the data recorded by the patient in symptom diaries with their health professional and will generate an alarm if a serious side effect occurs [13].

Moreover, computer-assisted history-taking systems (CAHTS) are other eHealth tools that have the potential to improve the monitoring of drug interactions and side-effects. CAHTS allow patients to enter their medication history prior to consultations, resulting in a more comprehensive record of medication information [14].

While there are several existing eHealth technologies for drug-drug interactions and stand-alone drug adverse effects, it appears that considerably less attention is focussed on CAM-drug interactions, CAM-CAM interactions, and standalone CAM adverse effects. Despite poor knowledge of their potential interactions and side effects, many patients use CAM. This justifies the need to identify what eHealth technologies exist for such CAM-related interactions and adverse effects.

## Methods

### Approach

A scoping review was conducted to identify eHealth technologies assisting in identifying potential adverse interactions with CAM or standalone CAM adverse events or side effects, following Arksey and O’Malley’s [15] five-stage scoping review framework, and supplemented by Levac, Colquhoun, & O’Brien [16] and Daudt, van Mossel, & Scott [17] which build upon Arksey and O’Malley’s work. The five steps are as follows: (1) identifying the research question, (2) identifying relevant studies, (3) selecting the studies, (4) charting the data, and (5) collating, summarizing, and reporting the results. This method was chosen in order to fulfill the prerequisites of a scoping review, which involve searching for and assessing the available literature on a given topic in order to identify the characteristics of eligible articles, summarize their contents and highlight knowledge gaps. We did not register a protocol.

### Step 1: identifying the research question

The research question for this scoping review was as follows: “What eHealth technologies are assisting in identifying potential 1) adverse drug interactions with CAM, 2) adverse CAM-CAM interactions or 3) standalone CAM adverse events or side effects?”. While a multitude of definitions for “eHealth” exists [18], for the purpose of this scoping review, in order to define parameters of eHealth, we considered 3 domains and their subcategories based on a study by Shaw et al. [19]. The framework for defining eHealth technologies are summarized in Table 2 in Appendix 3 of their paper. CAM has also been defined in a multitude of ways [20], however, the National Center for Complementary and Integrative Health (NCCIH) defines a non-mainstream practice used together with conventional medicine as “complementary”, a non-mainstream practice used in place of conventional medicine as “alternative”, and the coordinated delivery or use of conventional and complementary approaches as “integrative” [21]. For the purpose of this scoping review, in order to define parameters of eHealth, we considered Wieland et al.’s [22] bibliometric and content analysis of the Cochrane Complementary Medicine Field specialized register of controlled trials, where the authors collected the number of CAM field specialized register citations classified by type of CAM therapies. What was included as CAM are shown in Table 4 in Appendix 3 of their paper. Finally, we define the term “adverse event” as “any untoward medical occurrence that may present during treatment with a medicine but which does not necessarily have a causal relationship with this treatment”, and “side effect” as “any unintended effect of a pharmaceutical product [or CAM] occurring at doses normally used in man, which is related to the pharmacological proprieties of the drug [or CAM]”. Our definition of the terms “adverse event” and “side effect” correspond with that of the World Health Organization [23].

### Step 2: finding relevant studies

Following a preliminary scan of the literature, an experienced academic librarian was consulted to assist in devising a comprehensive, systematic search strategy on MEDLINE, EMBASE, and AMED academic databases. The search included literature published from 1995 up until November 6, 2019, as eHealth was only popularized in the late 1990s with the term itself was coined in 1999 [24]. The search strategy included Medical Subject Headings and keywords that reflect terms commonly used in the literature to refer to both eHealth and CAM. Following preliminary searches, it was decided not to also include search terms relating to adverse events or side effects, as many eligible articles were found to not be indexed using them and thus this would have excluded them. Additionally, the Canadian Agency for Drugs and Technologies in Health (CADTH) website (https://www.cadth.ca/) was also searched to account for any grey literature; terms searched included “eHealth”, “mHealth”, “complementary and alternative medicine” and “herbal”. A search strategy we used including Medical Subject Headings and keywords that reflect terms commonly used in the literature to refer to CAM and eHealth can be found in Appendix 1.

### Step 3: selecting the studies

Preliminary searches indicated that the academic literature on this subject area exists as eligible articles could be found. We included primary research articles and research protocols; any relevant reviews were used to source additional eligible primary research articles or research protocols. In order to be included, the article had to have included an eHealth technology (either the authors’ own or referenced) of any kind that was assisting in identifying potential 1) adverse drug interactions with CAM, 2) adverse CAM-CAM interactions, or 3) standalone CAM adverse events or side effects, otherwise they were excluded. At this stage, articles were excluded if they did not make reference to our research question. Publications in the form of conference abstracts were not eligible. We also restricted eligibility to articles published in the English language and that were either publicly available or could be ordered through our library system. If there was any uncertainty, the article’s full-text was reviewed to determine eligibility. We placed no eligibility restrictions on type of eHealth technology; even if they were only accessible in a non-English language, we included them as long as English literature was written about them. All three authors (JYN, MM, and VM) pilot-screened a subset of all titles and abstracts independently and met to verify their agreement in applying the inclusion criteria prior to screening all items, including the full-texts of potentially eligible articles, independently in triplicate. Disagreement was solved by discussion, and in the case that consensus could not be reached, a majority vote was used to determine eligibility.

### Step 4: charting the data

Articles meeting the inclusion criteria were critically reviewed using Arksey and O’Malley’s [15] descriptive-analytical narrative method. For each eligible article, the following data were extracted and charted: article title; author(s); year of publication; study country; study design; whether the article was original research or a review of resources; study aim; and name of eHealth technology(s) assessed or referenced in the eligible article that we assessed in this scoping review. For each included eHealth technology, the following data were extracted and charted: name; URL (if available), type (i.e. adverse drug reporting system, database, factsheets, etc.), format (i.e. website, mobile app, etc.), year established; if the eHealth technology still exists; whether it has been used in any context outside of the authors’ study; whether it is free and/or available to anyone; developer (and category); purpose; and intended user(s). All three authors (JYN, MM, and VM) participated in a pilot data extraction of a subset of eligible articles/eHealth technologies, and MM and VM independently extracted data from all eligible articles as well as from all eHealth technologies (i.e. the authors’ own or referenced). All three authors then met to discuss and resolve discrepancies. We did not conduct a critical appraisal of included sources of evidence nor did we collect included articles’ sources of funding, as no prior scoping review had been conducted on this topic before, thus we only aimed to provide a descriptive map of the literature and highlight a number of key themes that emerged from our analysis.

### Step 5: collating, summarizing, and reporting the results

Charted data was summarized in the format of tables, and the descriptive data were analysed using content analysis. All three authors (JYN, MM, and VM) reviewed the descriptive data, and JYN identified codes relative to the findings, organized codes into thematic groups, and presented a narrative relating to the research question as well as highlighted knowledge gaps in the currently existing literature. All three authors then met to discuss and resolve discrepancies.

## Results

### Search results

**Fig. 1:**
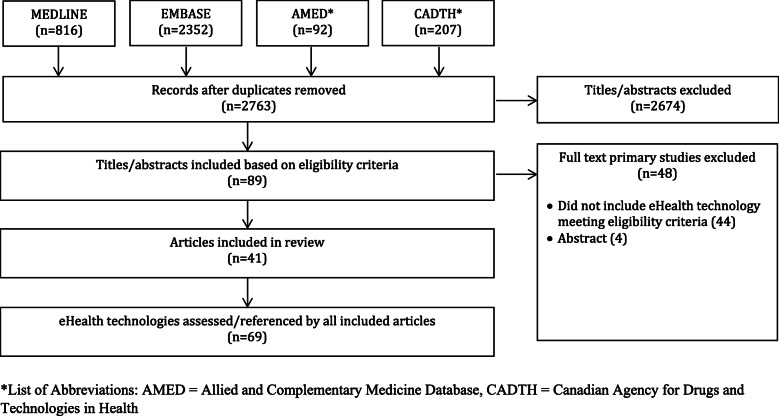
PRISMA Diagram. *List of Abbreviations: AMED = Allied and Complementary Medicine Database, CADTH = Canadian Agency for Drugs and Technologies in Health

Searches identified a total of 3467 items, of which 2763 were unique, and 2674 titles and abstracts were eliminated, leaving 89 full-text articles to be considered. Of those, 48 were not eligible, because they did not include eHealth technology meeting eligibility criteria (n=44) or were an abstract (n=4), leaving a total of 41 articles that were included in this scoping review [25–65]. A PRISMA diagram can be found in Fig. 1 of Appendix 2.

### Eligible article characteristics

Eligible articles were published from 1997 to 2019 and originated from the United States (n=16), China (n=8), Germany (n=3), Republic of Korea (n=3), Singapore (n=3), Canada (n=1), Denmark (n=1), Greece (n=1), Italy (n=1), Sweden (n=1), and United Kingdom (n=1). Additionally, one study involved researchers from Australia, China, and Germany (n=1), and another from China and the United Kingdom (n=1). Of the 41 articles included, 27 were primary research articles with the following study designs: development (n=12) or evaluation (n=7) of an eHealth technology, analysis of data collected by an eHealth technology (n=7), and a usability study (n=1); the remaining 14 articles were reviews of one or more medical information resource(s) including at least one containing an eHealth technology addressing our research question. Here, we define a “review” as an article that reviewed an aspect of the entirety of an eligible eHealth technology, and not necessarily a systematic or scoping review. The details associated with all eligible article characteristics, including study aims, can be found in Table 1 of Appendix 3.

### eHealth technology characteristics

Of the 69 included eHealth technologies, we characterized them as follows: databases (n=34), factsheets/healthcare information (n=13), adverse drug/event alerting, reporting and/or signal detection systems (n=11), search engines (n=4), interaction checkers (standalone) (n=1), bulletin (n=1), continuing education module (n=1), electronic pharmacovigilance system (n=1), model (n=1), and serious game (n=1). Additionally, one eHealth technology is both an adverse drug reaction detection/spontaneous reporting system and a database. These eHealth technologies were offered in the following formats: websites/web-based only (n=38), website and mobile app (n=10), mobile app only (n=6), software (n=2), artificial intelligence (n=1). The format was unclear for the remaining twelve. In many cases, it was difficult to ascertain the year the eHealth technology was created (n=29), however, 15 were identified to have been created from at least the mid-1970s to 2000, another 15 from 2001 to 2010, and 10 from 2011 to 2019. Forty-eight eHealth technologies were identified to currently exist, while 2 did not, and 1 was replaced by one of the 48, and for the remaining 18, their current existence was unclear. We found that 60 eHealth technologies had been used in any context outside of the authors’ study; for example, the technology or its data were cited in another research article not conducted by the developing authors. Thirty-two eHealth technologies were found to be available and entirely free to use by anyone, 12 are only accessible with a subscription, 1 was partially available without a subscription, required a partial-subscription, 3 were confirmed to be not available to the general public, and the status of the remaining 21 was unclear. eHealth technologies were developed by the following: companies (n=24), researchers or research groups (n=22) (11 of which by the authors of one or more of the eligible articles), government agencies/departments (n=17), botanical council (n=1), not-for-profit (n=1), practitioners (n=1), unclear (n=1). Additionally, 2 were developed by organizations that involved both researchers and government. Intended users of the eHealth technologies were not always clear, and we used our discretion in cases where this information was not provided explicitly. Our assessment was as follows: for healthcare providers (n=54), for researchers (n=36), for patients/public (n=32). These numbers reflect a large amount of overlap among these user types across many of the eHealth technologies. The details associated with all included eHealth technologies can be found in Table 2 of Appendix 3.

### Findings from thematic analysis

In total, three main themes emerged from our analysis and are described below.

### Obsolete reviews of CAM-related healthcare information

Upon accounting for all eligible articles, one immediate and striking finding was the fact that while a number of reviews have been published providing overviews or summaries of CAM-related healthcare or research resources, they have all been published in excess of one decade ago, with the exception of Xie et al. [41]‘s review, however it is only specific to natural product databases. The most recent reviews providing information on CAM, in general, were published approximately 15–20 years ago [25, 28, 32, 33, 48, 50, 56, 65]. As a result, the information contained within these articles is all, to varying extents, obsolete, including resources that are no longer available or updated. Furthermore, as these articles were published years before the methodologies of systematic or scoping reviews were published, many resources included in these reviews were found unsystematically or based on the authors’ knowledge.

### Large number of databases

It was found that a large number of databases exist, primarily offered in website and/or mobile app formats, making up the largest category of types of eHealth technology in this scoping review (35 of 69). New databases have been created (and maintained) since the 1970s up to the present day, and their developers and content vary fairly widely. Perhaps unsurprisingly, the vast majority of databases developed by companies focus on the provision of resources for healthcare providers. These include a number of well-recognized, large companies in the healthcare industry, providing items such as monographs, clinical decision suites, dosing calculators and adverse event/interaction checkers in their respective databases [66–71]. In contrast, databases developed by government organizations or agencies collect information at the population level regarding pharmacovigilance and adverse events [49, 72, 73], though databases such as the Drug Product Database available on the Health Canada website also provides a search tool for drugs and certain natural health products available nationally with associated monographs. Interestingly, most databases developed by researchers or research groups emerge from Asia (notably China and the Republic of Korea). These databases serve a different purpose than the aforementioned company- and government-developed ones, instead housing information on traditional medicine (i.e. traditional Chinese medicine) ingredients such as interactions, mechanisms of action, compound structures, and their relationship to genes and diseases [30, 31, 39–41, 47, 55].

We did not formally assess the usability of these databases or the quality of the information contained within them, as this exceeded the scope of this scoping review. Despite this, it should be noted that initial impressions obtained simply by completing the data extraction step for this review indicated that these databases likely vary largely in quality, and the authors intend on assessing this in a future research study. The frequency of updates for each database is presented in Table 3 in Appendix 3; some of the most recent databases (updated and/or created within the last 3 years) are presented in Table 4 in Appendix 3.

### Government adverse drug/event surveillance

We identified that the majority of adverse drug/event surveillance eHealth technologies including CAM identified were government initiated, and found across the following countries: China, India, the Republic of Korea, and the United States. These eHealth technologies are equipped with detection [34] and reporting systems [27, 31, 34, 74] and safety alerts [74, 75]. The countries with such government initiatives outside of the United States are unsurprising, given that a very large proportion of traditional and indigenous medical systems originate from these parts of the world, notably traditional Chinese and Korean medicine and Ayurvedic medicine [76]. Though a not-for-profit, as opposed to a government agency, our review also captured the Institute for Safe Medication Practices in Canada which runs a medication error reporting program and publishes newsletters, reports and safety alerts based on information received [77]. These government initiatives highlight the growing need to collect information surrounding CAM-related adverse events and side effects given they are widely used across the globe [78].

## Discussion

The purpose of the present scoping review was to identify eHealth technologies that assist in identifying potential 1) adverse drug interactions with CAM, 2) adverse CAM-CAM interactions, or 3) standalone CAM adverse events or side effects. The amount of available literature on this topic as well as the number of eHealth technologies, while not overly voluminous, presents a broad range of different eHealth technologies that have emerged since the popularization of (and even before) the term “eHealth”. Given that, to our knowledge, this is the first study to present such eHealth technologies using a systematic search of the peer-reviewed and grey literature, it is hoped that these findings will provide both healthcare providers and researchers with an awareness of what research has taken place over the past few decades at the intersection of CAM and eHealth.

### Resources for practitioners, researchers and patients: ample, but of unclear quality

This review also provides readers with a list of currently existing resources that they may not have been aware of to date, which may aid in their clinical practice or research. While these resources have been developed, evaluated, studied or assessed, at least to some degree, by academic researchers, this review was only designed to scope out the number of eHealth technologies and their key characteristics. Unfortunately, we did not find that any authors of included articles expressed an intention to create new upcoming eHealth technologies in this topic area, beyond improving their own existing resource. As expected, our scoping review captured some well-known and authoritative resources, such as Natural Medicines [69], MicroMedex [67] and the NCCIH’s Herbs at a Glance [79], however, some others may be less known and their quality may be questionable. Therefore, we encourage users of any of these resources to exercise caution and use their professional judgement when utilizing any of these resources, especially those which may be unfamiliar to them.

### Areas identified for future research

We have identified a couple of areas for future research based on our findings.

Today there exists more information on the Internet about CAM and CAM-related adverse events and side effects than ever before, yet the quality of much of it is arguably questionable [80]. Clinicians, researchers, governments and policymakers, and patients alike all need resources that provide them with reliable, trustworthy, and current information. Combined with our finding that the vast majority of reviews on CAM-related healthcare or research resources are now decades old, this undoubtedly justifies a need for an updated review of CAM-related healthcare information given that many changes have occurred in the way in which both guidelines inform clinical practice and research methodologies have evolved.

While we did not formally assess the quality of the eHealth technologies (nor the information they contained), it was evident at face-value that they varied across eHealth technologies across all types, developers and content areas. We hypothesize that this may relate to the fact that little guidance exists in developing or evaluating CAM-related eHealth technologies, as the growth of research conducted in this area has been slow and the information limited. While there have been discussions surrounding the creation of comprehensive information resources about CAM, it appears that the most recent ones took place nearly two decades ago [81, 82], based on preliminary searches we conducted prior to finalizing our systematic search strategy. The development of CAM-related eHealth technologies should be viewed as a research method in itself similar to how guidelines for different study types exist [83]. As a result, additional and updated guidance documents are needed in creating eHealth technologies, let alone CAM-related ones, as to date only one appears to exist [84].

### Strengths and limitations

Notable strengths of this study included the use of a comprehensive systematic search strategy to identify eligible articles, devised with the assistance of an experienced academic librarian. Interpretation of these findings was strengthened by the fact that all three authors independently screened, data extracted, and summarized the findings.

Limitations include the fact that this scoping review did not include non-English language articles, which perhaps would have been of importance given our finding that there is an emergence of new CAM-related eHealth technologies in Asia, among other countries. Additionally, it should be noted that many apps exist that likely also detect CAM-related adverse events or side effects which were not captured by this search. Though this is a limitation, it can be inferenced that higher-quality apps (or at least those that have been exposed to peer-review) would be those that would be captured in the published literature, which would be of greater use to healthcare providers and researchers as the primary audience of this review. Furthermore, a very large number of poor-quality apps exist on platforms such as the Google Play Store and the Apple App Store, and it would not be practical nor feasible to review them all.

## Conclusion

The present scoping review involved a systematic search of the literature to identify eHealth technologies assisting in identifying potential 1) adverse drug interactions with CAM, 2) adverse CAM-CAM interactions, or 3) standalone CAM adverse events or side effects. Having identified 69 unique eHealth technologies that fall into this category, we provide a descriptive map of the literature on this area and highlight a number of key themes that emerged from our analysis. Additionally, we highlight that while an ample number of resources are available to healthcare providers, researchers, and patients, we caution that the quality and update frequency for many of these resources vary widely, and until formally assessed, remain unknown. Lastly, we identify that a need exists to conduct an updated and systematically-searched review of CAM-related healthcare or research resources, as well as develop guidance documents associated with the development and evaluation of CAM-related eHealth technologies.

## Data Availability

All data generated or analysed during this study are included in this published article [and its supplementary information files].

## Appendix 1

### Sample Search Strategy

**MEDLINE Search Strategy for Scoping Review of eHealth Technologies Assisting in Identifying Potential 1) Adverse Drug Interactions with CAM, 1) Adverse CAM-CAM Interactions or 3) Standalone CAM Adverse Events or Side Effects Executed November 6, 2019**

**Table:**

Database: Ovid MEDLINE(R) and Epub Ahead of Print, In-Process & Other Non-Indexed Citations, Daily and Versions(R) <1946 to November 05, 2019> Search Strategy:	
1 ((Alternative or Traditional or Complementary or Integrat*) adj2 (Therap* or Medicine*)).mp. [mp=title, abstract, original title, name of substance word, subject heading word, floating sub-heading word, keyword heading word, organism supplementary concept word, protocol supplementary concept word, rare disease supplementary concept word, unique identifier, synonyms] (103649) 2 Complementary Therapies/ or Integrative Medicine/ or exp Medicine, East Asian Traditional/ or exp Medicine, Chinese Traditional/ or exp Herbal Medicine/ or exp Plants, Medicinal/ or exp Phytotherapy/ or exp Drugs, Chinese Herbal/ or exp Medicine, Ayurvedic/ (151984) 3 (CAM or TCM or Traditional Chinese Medicine or Ayurved* or Medicinal Plant* or Herbalism).mp. (69971) 4 (Herb* adj1 (Medic* or Therap* or Supplement*)).mp. (23276) 5 or/1-4 (248865) 6 (Telemedicine or Telehealth or eHealth or e-Health or mHealth or m-Health or Mobile Health or Health Records, Personal or Mobile Application* or E-Prescription* or Electronic Prescri* or Electronic Health Record* or Electronic Medical Record* or Medical Records System* or Health Informatics or Medical Informatics or Computeri?ed Decision Support or Data Mining or Decision Support System* or Wearable Electronic Device* or Wearable Technolog* or Smartphone or Iphone or I-phone or Android or Handheld Computer or Personal* Digital or Deep Learning or Artificial Intelligence).mp. (166979) 7 Telemedicine/ or Health Records, Personal/ or Mobile Applications/ or Electronic Prescribing/ or Medical Records Systems, Computerized/ or Medical Informatics/ or Drug Therapy, Computer-Assisted/ or Drug Information Services/ or Hospital Information System/ or Computing Methodologies/ or Wearable Electronic Devices/ or Artificial Intelligence/ or "Neural Networks (Computer)"/ (112744) 8 or/6-7 (199016) 9 5 and 8 (1157) 10 limit 9 to (english language and yr="1995 -Current") (815) ***************************	

## Appendix 3

### Data Extraction Tables

Table 1

**Table 1:** Eligible article characteristics (*n* = 41)

First Author and Year	Article title	Study Country	Study Design	Article type	Study aim
ORIGINAL RESEARCH ARTICLES (*n* = 27)					
Archer et al. 2014 [35]	Development of an alert system to detect drug interactions with herbal supplements using medical record data	USA	Development of alert system prototype	Original Research	To develop an automated herb-drug interaction alert system prototype designed to alert physicians and patients of potential herb-drug interactions
Boehmer et al. 2011 [53]	Evaluating the value of a web-based natural medicine clinical decision tool at an academic medical center	USA	Evaluation of web-based clinical decision tool	Original Research	To evaluate the impact that a natural medicine clinical decision tool has on faculty attitudes, practice experiences, and needs with respect to herbal and natural products
Brink et al. 2004 [60]	Cancer CAM(TM): Web-based continuing education for health professionals	USA	Evaluation of online continuing education prototype module	Original Research	To describe “a formative evaluation of a web-based continuing education program for nurses and patient health educators on CAM for cancer patients” (p. 44)
Chen et al. 2002 [29]	Computer automated prediction of potential therapeutic and toxicity protein targets of bioactive compounds from Chinese medicinal plants	Singapore	Evaluation of software	Original Research	To determine the therapeutic and toxicity protein targets of Chinese medicinal plant compounds
Chen et al. 2006 [30]	Database of traditional Chinese medicine and its application to studies of mechanism and to prescription validation	China	Development of database and artificial intelligence systems	Original Research	To collect quantitative information about traditional Chinese medicine (TCM) prescriptions, constituent herbs and herbal ingredients to create/test a database to assist with the studying and exploring of TCM
Ee et al. 2018 [64]	Herbopolis - A mobile serious game to educate players on herbal medicines	Singapore	Usability study for a mobile game prototype	Original Research	To develop a mobile game to motivate users to learn more about herbal medicine
Faubert et al. 2010 [54]	A pilot study to compare natural health product-drug interactions in two databases in Canada	Canada	Evaluation of databases	Original Research	To evaluate and compare two natural health product databases for the purpose of integrating them into a pharmacy information system in Canada
Fischer et al. 2005 [44]	Complementary and alternative medical reference software for personal digital assistants: evidence of clinical applicability	USA	Evaluation of databases	Original Research	To evaluate the value and clinical applicability of new complementary and alternative medicine software products
Fucik et al. 2002 [49]	Building a computerized herbal substance register for implementation and use in the World Health Organisation International Drug Monitoring Programme	Sweden	Development of database	Original Research	To build a computerized herbal substance register for implementation and use in the World Health Organisation International Drug Monitoring Programme
Gao et al. 2015 [59]	Pharmacovigilance in China: Issues of concern identified through an analysis of the Chinese Adverse Drug Reaction Information Bulletin 2001 to 2014	China	Analysis of Adverse Drug Reaction Information Bulletin (ADRIB) reports	Original Research	To analyse the reports in the ADRIB since its first publication in 2001 to give international readers a better appreciation of the pharmacovigilance issues addressed
Gregory et al. 2016 [42]	Characterization of complementary and alternative medicine-related consultations in an academic drug information service	USA	Analysis of complementary and alternative medicine drug information consultations	Original Research	“To evaluate and characterize consultation requests received through our academic drug information consultation service related to complementary and alternative medicines” (p. 540)
Hamre et al. 2017 [61]	Use and safety of anthroposophic medicinal products: An analysis of 44,662 patients from the EvaMed Pharmacovigilance Network	Germany	Analysis of EvaMed Pharmacovigilance Network diagnoses and prescriptions data	Original Research	To determine the frequency of adverse drug reactions to anthroposophic medicinal products (AMPs), relative to the number of AMP prescriptions.
Kim et al. 2019 [45]	Drug repositioning of herbal compounds via a machine-learning approach	Republic of Korea	Development of algorithm	Original Research	“To predict new indications for existing drugs and additional herbal compounds based on a machine-learning approach” (p. 34)
Lee et al. 2015 [40]	PharmDB-K: Integrated bio-pharmacological network database for traditional Korean medicine	Republic of Korea	Development of database	Original Research	To construct PharmDB-K, which offers comprehensive information relating to Traditional Korean Medicine-associated drugs (compound), disease indication, and protein relationships
Li et al. 2009 [26]	A web-based quantitative signal detection system on adverse drug reaction in China	China	Development of signal detection system	Original Research	“To establish a web-based quantitative signal detection system for adverse drug reactions based on spontaneous reporting to the Guangdong province drug monitoring database in China” (p. 729)
Ogultarhan et al. 2016 [36]	KATIS: An eHealth system for complementary medicine	Germany	Development of database and mobile app	Original Research	To aggregate knowledge on complementary and alternative medicine (CAM) into one database to allow for the search of CAM therapies, indications and interactions
Olesen et al. 2013 [43]	Absence of ‘over-the-counter’ medicinal products in on-line prescription records: A risk factor of overlooking interactions in the elderly	Denmark	Analysis of online prescription record data	Original Research	“To assess possible origins of harmful interactions in elderly patients arising from the current absence of information on over-the counter (OTC) medicines in the Danish ‘on-line prescription record’” (p. 145)
Spanakis et al. 2019 [46]	PharmActa: Empowering patients to avoid clinical significant drug-herb interactions	Greece	Evaluation of mobile app	Original Research	To discuss the use of personal health services and mobile health applications for patients and healthcare providers to avoid and manage drug-herb interactions, and to discuss a recently developed personalized pharmaceutical mobile health application called PharmActa
Sun et al. 2019 [55]	Development of quantitative structure-activity relationship models to predict potential nephrotoxic ingredients in traditional Chinese medicines	China	Development and testing of model	Original Research	To develop a quantitative structure-activity relationship models to predict potential nephrotoxic ingredients in traditional Chinese medicines
Tabali et al. 2012 [62]	Adverse drug reactions for CAM and conventional drugs detected in a network of physicians certified to prescribe CAM drugs	Germany	Analysis of adverse drug reactions in database	Original Research	“To describe and quantify the volume and severity of adverse drug reactions for complementary and alternative medicine (CAM) and conventional drugs in a proprietary database created from prescriptions and patient data of primary care CAM physicians who participate in the EvaMed Network” (p. 427)
Walker 2002 [51]	Evaluation of the ability of seven herbal resources to answer questions about herbal products asked in drug information centers	USA	Evaluation of databases	Original Research	“To evaluate the ability of seven widely known herbal references and electronic databases to answer questions about herbal products asked at drug information centers” (p. 1611)
Woo et al. 2019 [31]	Safety of herbal medicine for elderly patients with chronic disease in the Republic of Korea	Republic of Korea	Analysis of spontaneous adverse event reports	Original Research	To investigate the detection of drug safety signals associated with herbal medicine by analyzing spontaneous adverse event reports in elderly patients with chronic diseases to generate new safety information
Xu et al. 2019 [47]	ETCM: An encyclopaedia of traditional Chinese medicine	China	Development of web-based encyclopedia	Original Research	To develop an online encyclopedia to provide users information on traditional Chinese medicine herbs and formulas
Yao et al. 2019 [63]	An ontology-based artificial intelligence model for medicine side-effect prediction: taking traditional Chinese medicine as an example	Australia, China, Germany	Development of artificial intelligence	Original Research	To develop an ontology-based model for artificial intelligence-assisted medicine side-effect prediction, and validate a proposed model consisting of three main components, including the drug model, the treatment model, and the artificial intelligence-assisted prediction model
Yap et al. 2012 [39]	Utilizing mobile networks for the detection of clinically relevant interactions between chemotherapy regimens and complementary and alternative medicines	Singapore	Development of an iPhone app	Original Research	“To develop a novel database application for the Mobile Internet called OncoRx-MI, the purpose of which is to detect drug-complementary and alternative medicine interactions of both single-agent and multiple-agent chemotherapy regimen prescriptions” (p. 166)
Ye et al. 2009 [34]	A computerized system for signal detection in spontaneous reporting system of Shanghai China	China	Development of signal detection system and reporting system	Original Research	To develop a computerized signal detection system to detect adverse drug reactions
Zhang 2018 [27]	Pharmacovigilance of herbal and traditional medicines	China	Analysis of pharmacovigilance of herbal and traditional medicines	Original Research	To differentiate the concepts of traditional/complementary medicine and their products, and introduce the supervision and management systems of the China Food and Drug Administration and the differences between conventional medicine and traditional/complementary medicine products, taking drugs used in traditional Chinese medicine as an example
REVIEWS (*n* = 14)					
Allais et al. 2000 [65]	Access to databases in complementary medicine	Italy	Review of medical information resource(s)	Review	To review and categorize biomedical databases for complementary and alternative medicine
Boddy et al. 2008 [52]	Review of reliable information sources related to integrative oncology	UK	Review of medical information resource(s)	Review	“To provide an overview of reliable integrative oncology information from various resources.” (p. 620)
Clauson et al. 2008 [37]	Clinical decision support tools: Personal digital assistant versus online dietary supplement databases	USA	Review and evaluation of databases and personal digital assistants	Review	To “assess and compare the content of PDA dietary supplement databases and their online counterparts used as clinical support decision tools” p. 1593
Fitzpatrick 2010 [38]	Natural standard database	USA	Review of medical information resource(s)	Review	To “provide an overview of Natural Standard and its content and scope, as well as provide some basics for searching this resource”(p. 154)
Jackson 2001 [50]	An overview of information resources for herbal medicinals and dietary supplements	USA	Review of medical information resource(s)	Review	To “provide a comprehensive, annotated listing of reliable resources of information on herbs and dietary supplements divided into the following categories: journals, databases and websites, and books and compendia” (p. 36)
Jackson et al. 2001 [25]	Resources for information on herbal medicinals and dietary supplements	USA	Review of medical information resource(s)	Review	To assess information on herbal medicinals and dietary supplements on AltMedDex
Kiefer et al. 2001 [32]	Finding information on herbal therapy: a guide to useful sources for clinicians	USA	Review of medical information resource(s)	Review	To provide primary care clinicians with a list of general, clinically oriented, evidence-based, English language references for Western herbal therapeutics that may be of practical use in the clinical setting
Meyer et al. 2004 [33]	Evaluation of herbal-drug interaction data in tertiary resources	USA	Review of medical information resource(s)	Review	To objectively evaluate various tertiary resources using a set of predetermined criteria to assess which provide the most complete, current, and accurate herbal-drug interaction information
Molassiotis & Zu 2004 [58]	Quality and safety issues of web-based information about herbal medicines in the treatment of cancer	China, UK	Review of medical information resource(s)	Review	To assess the quality and safety of the information presented on the internet about medicinal herbs specifically in the field of cancer
Motl et al. 2004 [28]	Health information websites by therapeutic category for healthcare professionals	USA	Review of medical information resource(s)	Review	“To compile and evaluate health information websites to aid healthcare professionals in locating information on select therapeutic categories” (p. 106)
Sweet et al. 2003 [56]	Usefulness of herbal and dietary supplement references	USA	Review of medical information resource(s)	Review	“To describe the usefulness of some of the most common tertiary references that healthcare professionals employ to answer requests about herbal and dietary supplements” (p. 494)
Tomasulo 2003 [57]	Natural Standard--New integrative medicine database	USA	Review of medical information resource(s)	Review	To provide an overview of the Natural Medicines (Natural Standards) database
Wootton 1997 [48]	Directory of databases for research into alternative and complementary medicine: An update	USA	Review of medical information resource(s)	Review	To provide a directory of databases for research into complementary and alternative medicine
Xie et al. 2015 [41]	Review of natural product databases	China	Review of medical information resource(s)	Review	To provide an overview and analysis of current natural product databases and discuss trends of future database development

Table 2

**Table 2:** eHealth technology characteristics (*n* = 69)

Name of eHealth Technology	Source Eligible Article(s) Included in Review	URL of eHealth Technology?	Type of eHealth Technology	Format	Year Established	Still in Existence?	Used Outside of the Authors’ Study?	Available for Anyone to Use?	Developer of eHealth Technology	Type of Developer	Purpose of eHealth Technology	Intended User(s) of eHealth Technology
Adverse Drug/Event Alerting, Reporting and/or Signal Detection Systems (*n* = 11)												
Center for Food Safety and Applied Nutrition	Jackson et al. 2001 [25]	https://www.fda.gov/about-fda/office-foods-and-veterinary-medicine/center-food-safety-and-applied-nutrition-cfsan	Adverse event reporting tool and safety alerts	Website	1984	Yes	Yes	Yes, entirely free	U.S. Food and Drug Administration	Government	Website containing safety alert publications, guide documents and a reporting tool for dietary supplement adverse events	Healthcare providers, researchers, patients/public
Guangdong Quantitative Signal Detection System (GDQSDS)	Li et al. 2009 [26]; Zhang 2018 [27]	No	Adverse drug reaction signal detection and spontaneous reporting system	Web-based	Unclear	Unclear	Yes	Unclear	Authors	Researchers (authors)	“A web-based system comprising three software modules that prepare data, detect associations, and generate reports, was developed based on the Guangdong ADR monitoring platform” p. 730	Researchers
Institute for Safe Medication Practices (ISMP)	Motl et al. 2004 [28]	https://www.ismp.org	Adverse event reporting system	Website	1995 (website)	Yes	Yes	Yes, entirely free	The Institute for Safe Medication Practices (ISMP)	Not-for-Profit	Contains a medication error reporting program, and publishes newsletters, reports and safety alerts based on information received	Healthcare providers, patients/public
INVDOCK	Chen et al. 2002 [29]; Chen et al. 2006 [30]	Unclear	Adverse drug reaction signal detection	Software	2001	Yes	Yes	Unclear	Chen & Zhi (2001). See: 10.1002/1097-0134(20010501)43:2%3C217::AID-PROT1032%3E3.0.CO;2-G	Researchers	Software that allows for “computer-aided identification of potential protein targets of a small molecule” (Chen & Zhi 2001, p. 225 [85])	Researchers
Korea Institute of Drug Safety and Risk Management Korea Adverse Event Reporting System database (KIDS-KD)	Woo et al. 2019 [31]	Unclear	Adverse event reporting system and database	Unclear	2012	Unclear	Yes	Unclear	Korean Institute of Drug Safety & Risk Management (KIDS)	Researchers/Government	An adverse drug reaction reporting system	Healthcare providers
MedWatch	Jackson et al. 2001 [25]; Kiefer et al. 2001 [32]; Meyer et al. 2004 [33]; Motl et al. 2004 [28]	https://www.fda.gov/safety/medwatch-fda-safety-information-and-adverse-event-reporting-program/medical-product-safety-information	Adverse event reporting tool and safety reports	Website	Unclear	Yes	Yes	Yes, entirely free	U.S. Food and Drug Administration	Government	Safety alerts on drugs, natural products and other medical products	Healthcare providers, researchers, patients/public
National Coordination Centre, Pharmacovigilance Programme of India (NCC-PvPI) Database	Zhang 2018 [27]	No	Adverse drug reaction reporting system	Unclear	2010	Yes	Yes	Unclear	Government of India	Government	To conduct traditional medicines surveillance though a drug adverse event reporting system which reports to Vigibase	Healthcare providers, researchers, health organizations, patients/public
Shanghai Adverse Drug Reaction Spontaneous Reporting System (ADR-SRS)	Ye et al. 2009 [34]	Unclear	Adverse drug reaction detection/spontaneous reporting system and database	Unclear	2001	Yes	Yes	Unclear	National Adverse Drug Reaction Monitoring Center of China	Government	Web-based ADR detection system and reporting tool for TCM and chemical medicine	Healthcare providers, pharmaceutical manufacturers, researchers
Shanghai Drug Monitoring and Evaluative System (SDMES)	Ye et al. 2009 [34]; Zhang 2018 [27]	Unclear	Adverse drug reaction surveillance system	Unclear	2001	Unclear	Yes	Unclear	Shanghai Center for Adverse Drug Reaction Monitoring	Government	To locally monitor marketed drugs (including herbs) by working in partnership with ten hospitals in Shanghai that permit direct access to patient adverse drug reaction information	Researchers
Special Nutritionals Adverse Event Monitoring System	Jackson et al. 2001 [25]	Unclear	Adverse event reporting system	Web-based	Unclear	Unclear	Yes	Yes, entirely free	U.S. Food and Drug Administration	Government	Collects adverse event reports on dietary supplements, infant formulas, and medical foods from a variety of sources	Healthcare providers, researchers, patients/public
Unnamed	Archer et al. 2014 [35]	No	Adverse drug reaction alerting system	Unclear	Unclear	Unclear	Unclear	Unclear	Authors	Researchers (authors)	To automatically detect herb-drug interactions and classify their severity	Healthcare providers, researchers
Yellow Card Scheme	Ye et al. 2009 [34]	https://yellowcard.mhra.gov.uk/	Adverse event reporting system	Website	Unclear	Yes	Yes	Yes, entirely free	Medicines and Healthcare Products Regulatory Agency	Researchers/Government	A reporting tool that monitors the safety of healthcare products, including herbal products, in the UK	Healthcare providers (including pharmacists), patients/public
Databases (*n* = 34)												
ABDAMED	Ogultarhan et al. 2016 [36]	https://abdata.de/datenangebot/abdamed/	Database	Website	Unclear	Yes	Yes	Unclear	ABDATA Pharma Data Service	Company	“ABDAMED is a commercial pharmaceutical database, which contains approved drug-related data, such as active ingredients, excipients, risks, indications, contraindications and adverse effects” (Ogultarhan et al. 2016, p. 169 [36])	Healthcare providers, researchers, patients
Alticopeia Herbal Medicine Database	Clauson et al. 2008 [37]; Fischer et al. 2005 [44]	http://www.ddhsoftware.com/gallery.html?show=number&record=527	Database	Mobile app	2001	Yes	Yes	Yes, entirely free	DDH Software	Company	“To facilitate understanding of herbal medicine by physicians and other ‘traditional’ health care workers.” (See: http://www.ddhsoftware.com/gallery.html?show=number&record=527)	Healthcare providers
ALTMEDA	Ogultarhan et al. 2016 [36]	Unclear	Database	Unclear	2016	Unclear	Unclear	Unclear	Authors	Researchers (authors)	A database that includes complementary and drug-related data (Ogultarhan et al. 2016, p. 167 [36])	Healthcare providers, researchers
American Botanical Council	Fitzpatrick 2010 [38]; Jackson et al. 2001 [25]; Kiefer et al. 2001 [32]; Motl et al. 2004 [28]	http://abc.herbalgram.org/site/PageServer	Database	Website	1988	Yes	Yes	Yes, partially without subscription	American Botanical Council	Council	A nonprofit, research and education organization, providing access to several databases (i.e. HerbMedPro, the Complete German Commission E Monographs) among other herbal resources (See: www.herbalgram.org)	Healthcare providers, researchers, patients/public
Caremark Drug Interactions	Yap et al. 2012 [39]	http://cpref.goldstandard.com/inter.asp?r=8084	Database	Website	Unclear	Yes	Yes	Yes, entirely free	Gold standard Inc.	Company	A tool for checking drug, herb and vitamin interactions	Patients/public
China Natural Products Database (CNPD)	Lee et al. 2015 [40]	Unclear	Database	Unclear	Unclear	Unclear	Unclear	Unclear	No information	Unclear	No information available	Researchers
Chinese Ethnic Minority Traditional Drug Database (CEMTDD)	Xie et al. 2015 [41]	http://www.cemtdd.com/ (inaccessible)	Database	Website	2015	Unclear	Yes	Unclear	Jinhui Wang	Researchers	A database containing Chinese minority herbs, built on data retrieved from various resources (mainly from Kazakh and Uygur traditional drugs) containing a variety of modules p. 399	Healthcare providers, researchers
Clinical Pharmacology (also known as Gold Standard)	Gregory et al. 2016 [42]	https://www.clinicalpharmacology.com/	Database	Website	Unclear	Yes	Yes	Yes, but only with a subscription	Elsevier	Company	Monographs, reports, guides, resources and drug information of prescription drugs and herbal/nutritional products	Healthcare providers, researchers
Danish National Drug Interaction Database	Olesen et al. 2013 [43]	http://www.interaktionsdatabasen.dk/	Database	Website	2003	Yes	Yes	Yes, entirely free	Danish Medicines Agency	Government	“The Danish Drug Interaction database is an electronic search tool for searching and learning about the effects of and adverse reactions from taking two or more different kinds of medication” (See: https://www.danishhealthdata.com/find-health-data/Interaktionsdatabasen)	Healthcare providers
DoubleCheckMD	Yap et al. 2012 [39]	Unclear	Database	Unclear	Unclear	No	Yes	Unclear	Enhanced Medical Decisions Inc.	Company	Tool for users to check for drug interactions and side effects	Healthcare providers, patients/public
DrDrugs	Clauson et al. 2008 [37]; Fischer et al. 2005 [44]	https://www.skyscape.com/product/drdrugs-drug-guide-for-physicians	Database	Website and mobile app	Unclear	Yes	Yes	Yes, but only with a subscription	Skyscape	Company	Drug information, dosing calculators, monographs, safety alerts	Healthcare providers
Drug Information (formerly DrugDigest)	Motl et al. 2004 [28]	https://www.express-scripts.com/medco/consumer/ehealth/druginfo/dlmain.jsp?WC=N	Database	Website	Unclear	Yes	Yes	Yes, entirely free	Express Scripts Inc	Company	To provide consumers with information on drugs and CAM supplements, including adverse effects	Patients/public
Drug Product Database	Motl et al. 2004 [28]	https://www.canada.ca/en/health-canada/services/drugs-health-products/drug-products/drug-product-database.html	Database	Website	Unclear	Yes	Yes	Yes, entirely free	Health Canada	Government	Search tool for drugs and certain natural health products in Canada containing monographs	Healthcare providers, patients/public
Drugbank	Kim et al. 2019 [45]; Lee et al. 2015 [40]; Spanakis et al. 2019 [46]	https://www.drugbank.ca/	Database	Website	2006	Yes	Yes	Yes, entirely free	Wishart Research Group	Researchers	A bioinformatics and cheminformatics resource that combines detailed drug data with comprehensive drug target information	Healthcare providers, researchers, patients/public
Encyclopedia of Traditional Chinese Medicine (ETCM)	Xu et al. 2019 [47]	http://www.nrc.ac.cn:9090/ETCM/	Database	Website	Unclear	Yes	Yes	Yes, entirely free	Authors	Researchers (authors)	Contains information and data on traditional Chinese medicine herbal drugs, formulas, predicted drug targets and diseases	Healthcare providers, researchers
Epocrates Rx (plus or pro)	Clauson et al. 2008 [37]; Fischer et al. 2005 [44]; Yap et al. 2012 [39]	https://online.epocrates.com/	Database	Website and mobile app	Unclear	Yes	Yes	Yes, but only with a subscription	Athenahealth	Company	Provides monographs, a drug interaction checker, pill identifier, dosing calculators, and formularies	Healthcare providers
Guide to Popular Natural Products	Clauson et al. 2008 [37]; Fischer et al. 2005 [44]	http://www.skyscape.com/EStore/ProductDetail.aspx?ProductID=951 (inaccessible)	Database	Mobile app	Unclear	No	Yes	Unclear	Skyscape	Company	No information available	Unclear
IBIS: Integrative BodyMind Information System	Allais et al. 2000 [65]; Kiefer et al. 2001 [32]; Wootton 1997 [48]	http://www.ouribis.com/	Database	Software	1992	Yes	Yes	Yes, but only with a subscription	Medicineworks	Company	Provides clinical reference database with drug-herb and drug-nutrient interactions, among other information about nutritional supplements and botanical preparations	Healthcare providers, researchers
International Drug Information System (INTDIS)	Fucik et al. 2002 [49]	No	Database	Unclear	1978	No (has been replaced by Vigibase)	Yes	No	World Health Organization International Drug Monitoring Program	Government	To collect and store information on herbal medicines, allopathic drugs and adverse drug reactions from various countries	National centers (i.e. World Health Organization), researchers, pharmaceutical companies
Lexi-Natural (includes LexiComp)	Clauson et al. 2008 [37]; Gregory et al. 2016 [42]; Fischer et al. 2005 [44]; Motl et al. 2004 [28]; Yap et al. 2012 [39]	http://webstore.lexi.com/Store/Individual-Databases/Lexi-Natural-Products	Database	Website and mobile app	Unclear	Yes	Yes	Yes, but only with a subscription	Wolters Kluwer	Company	Provides information on natural products, including interactions, adverse reactions, toxicology, etc. and includes monographs	Healthcare providers (including pharmacists, physicians, nurses, dentists), researchers
MedicinesComplete (includes Herbal Medicines; formerly known as the British National Formulary)	Jackson 2001 [50]; Jackson et al. 2001 [25]; Yap et al. 2012 [39]	https://about.medicinescomplete.com/publication/herbal-medicines/	Database	Website	Unclear	Yes	Yes	Yes, but only with a subscription	Pharmaceutical Press Editorial, The Royal Pharmaceutical Society	Company	Resources on herbal medicines including “uses, dosage, evidence of efficacy, adverse effects, contraindications, use in pregnancy and lactation, drug interactions” (See: https://www.pharmpress.com/product/MC_HERB/herbal-medicines)	Healthcare providers (including pharmacists)
Micromedex (includes DrugDex, Drug-Reax, AltMedDex)	Clauson et al. 2008 [37]; Fischer et al. 2005 [44]; Jackson 2001 [50]; Jackson et al. 2001 [25]; Kiefer 2001 [32]; Meyer et al. 2004 [33]; Walker 2002 [51]; Yap et al. 2012 [39]	https://www.micromedexsolutions.com/home/dispatch/ssl/true	Database	Website and mobile app	mid-1970s	Yes	Yes	Yes, but only with a subscription	IBM	Company	Contains evidence-based documents on prescription and non-prescription drugs; contains information such as dosing, adverse effects, interactions, warnings and efficacy	Healthcare providers, researchers
Natural Medicines (formerly Natural Medicine Comprehensive Database (NMCD) and Natural Standard Database (NSD))	Archer et al. 2014 [35]; Boddy et al. 2011 [52]; Boehmer et al. 2011 [53]; Clauson et al. 2008 [37]; Faubert et al. 2010 [54]; Fischer et al. 2005 [44]; Fitzpatrick 2010 [38]; Gregory et al. 2016 [42]; Jackson et al. 2001 [25]; Kiefer et al. 2001 [32]; Motl et al. 2004 [28]; Sun et al. 2019 [55]; Sweet al al. 2003 [56]; Tomasulo 2003 [57]; Walker 2002 [51]; Yap et al. 2012 [39]	https://naturalmedicines.therapeuticresearch.com/	Database	Website and mobile app	early 2000s	Yes	Yes	Yes, but only with a subscription	Therapeutic Research Center	Company	Contains information about herbs and dietary supplements, including 15 categories of information which address the most common questions faced by practitioners; also provides interactive tools for safety, effectiveness and interactions	Healthcare providers, researchers, patients
OncoRx Database (called Onco-Rx as a website, and OncoRx-MI as a mobile app)	Yap et al. 2012 [39]	http://www.onco-informatics.com/oncorx/	Database	Website and mobile app	2007 (website), unclear (mobile app)	Yes	Yes	Yes, but only with a subscription	Dr. Kevin Yap Research Group	Researchers	Detects drug-CAM interactions (DCIs) and provides information on “interaction effects, severities, mechanisms of interactions, substantiating evidences and references, as well as overall management plans for the chemotherapy regimens” (Yap et al. 2012, p. 167 [39])	Healthcare providers, researchers, patients/public
PEPID Drug Information Database	Clauson et al. 2008 [37]; Fischer et al. 2005 [44]	https://www.pepid.com/	Database	Website and mobile app	Unclear	Yes	Yes	Yes, but only with a subscription	PEPID	Company	Clinical decision suites for a variety of healthcare providers, providing information on drugs, herbal medicines and supplements, and an Interaction checker and toxicology resources	Healthcare providers
PharmDB-K	Lee et al. 2015 [40]	http://pharmdb-k.org/	Database	Website	Unclear	Yes	Yes	Yes, entirely free	Authors, Information Center for Bio-pharmacological Network (i-Pharm)	Researchers (authors)	Provides comprehensive traditional Korean medicine-associated compound, drug, disease indication, and protein relationship information	Researchers
PharmGKB	Yap et al. 2012 [39]	https://www.pharmgkb.org/	Database	Website	2001	Yes	Yes	Yes, entirely free	Shriram Center for Bioengineering and Chemical Engineering	Researchers	A pharmacogenomics knowledge resource providing clinical information including clinical guidelines and drug labels, potentially clinically actionable gene-drug associations and genotype-phenotype relationships	Healthcare providers (including pharmacists), researchers
Tarascon Pocket Pharmacopoeia	Clauson et al. 2008 [37]; Fischer et al. 2005 [44]	https://www.tarascon.com/products/mobile/	Database	Mobile app	Unclear	Yes	Yes	Yes, but only with a subscription	Tarascon Publishing	Company	Contains drug information to help clinicians make better decisions at the point of care	Healthcare providers
TCM Assistant	Yap et al. 2012 [39]	http://www.tcmassistant.com/ (inaccessible)	Database	Website	2004	Unclear	Yes	Unclear	e-MS Inc	Company	Provides resources on traditional Chinese medicine herbs, including herbs name, their formulas, their usage in curing disease and patent description	Healthcare providers, researchers
TCM Database@Taiwan	Lee et al. 2015 [40]; Sun et al. 2019 [55]; Xie et al. 2015 [41]	http://tcm.cmu.edu.tw/ (inaccessible)	Database	Website	2011	Unclear	Yes	Unclear	Calvin Yu-Chian Chen	Researchers	A database for Chinese traditional medicinal compounds, providing freely downloadable 3D compound structures of ingredients used in traditional Chinese medicine	Researchers
TCMGeneDIT	Xu et al. 2019 [47]	http://tcm.lifescience.ntu.edu.tw/ (inaccessible)	Database	Website	Unclear	Unclear	Yes	Unclear	Fang et al. 2018 See: 10.1186/1472-6882-8-58	Researchers	A traditional Chinese medicine database that provides information about genes, diseases, effects and ingredients	Researchers
TCM-MESH	Xu et al. 2019 [47]	http://mesh.tcm.microbioinformatics.org/	Database	Website	Unclear	Yes	Yes	Yes, entirely free	Zhang et al. 2017 See: 10.1038/s41598-017-03039-7	Researchers	Database that contains TCM-related information on herbs, compounds, diseases and genes. Includes side-effects and has a search tool	Healthcare providers, researchers
Traditional Chinese Medicine Information Database (TCM-ID)	Chen et al. 2006 [30]; Lee et al. 2015 [40]; Xu et al. 2019 [47]	http://bidd.nus.edu.sg/group/TCMsite/	Database	Unclear	2005	Yes	Yes	Yes, entirely free	Authors	Researchers (authors)	Provides information about traditional Chinese medicines including prescriptions, constituent herbs, herbal ingredients, molecular structure and functional properties of active ingredients, therapeutic and side effects, and clinical indications	Healthcare providers, researchers
Vigibase (includes Vigilyze)	Ye et al. 2009 [34]; Zhang 2018 [27]	https://www.who-umc.org/vigibase/vigibase/	Database	Website	1971	Yes	Yes	Yes, but only with a subscription	WHO Uppsala Monitoring Center, World Health Organization	Government	A global pharmacovigilance database of individual case safety reports, with over 20 million reports of suspected adverse effects of medicines	Healthcare providers (including physicians, dentists, nurses, and pharmacists)
Factsheets/Healthcare Information (*n* = 13)												
About Herbs	Boddy et al. 2008 [52]; Molassiotis et al. 2004 [58]; Yap et al. 2012 [39]	https://www.mskcc.org/cancer-care/diagnosis-treatment/symptom-management/integrative-medicine	Factsheets/healthcare information	Website and mobile app	Unclear	Yes	Yes	Yes, entirely free	Memorial Sloan-KetteringCancer Center	Researchers	Resources on herbs, botanicals and other CAM drugs. Includes information on CAM interactions	Healthcare providers, researchers, patients
ALMEKO	Ogultarhan et al. 2016 [36]	https://play.google.com/store/apps/details?id=de.icancode.almeko&hl=de	Factsheets/healthcare information	Mobile app	2016	Yes	Unclear	Unclear	Authors	Researchers (authors)	ALMEKO bundles information on different areas of complementary and alternative medicine, providing an indication check, and overview of potential therapies for the alleviation of symptoms (See: https://apkcombo.com/almeko/de.icancode.almeko/)	Healthcare providers, public
AlternativeDr.com	Motl et al. 2004 [28]	http://www.alternativedr.com	Factsheets/healthcare information	Unclear	Unclear	Unclear	Yes	Yes, entirely free	Advisory panel of MDs and experts in natural medicine	Practitioners	“Lists herbal interactions with several prescription and over the-counter medications” (Motl et al. 2004, p. 109 [28])	Healthcare providers, public
Complementary and Alternative Medicine	Boddy et al. 2008 [52]	https://www.cancer.gov/about-cancer/treatment/cam	Factsheets/healthcare information	Website	Unclear	Yes	Yes	Yes, entirely free	National Cancer Institute, National Institutes of Health	Government	Provides summaries on commonly used complementary and alternative medicines for patients and healthcare providers	Healthcare providers, patients/public
Dietary Supplement Fact Sheets	Motl et al. 2004 [28]	https://ods.od.nih.gov/factsheets/list-all/	Factsheets/healthcare information	Website	1998	Yes	Yes	Yes, entirely free	Office of Dietary Supplements, National Institute of Health	Government	Contains factsheets on supplements, vitamins and minerals	Healthcare providers, patients/public
Herb-Drug Interactions, NCCIH Clinical Digest	Allais et al. 2000 [65]; Boddy et al. 2008 [52]; Gregory et al. 2016 [42]; Jackson et al. 2001 [25]; Motl et al. 2004 [28]	https://nccih.nih.gov/health/providers/digest/herb-drug	Factsheets/healthcare information	Website	2015	Yes	Yes	Yes, entirely free	National Center for Complementary and Integrative Health (NCCIH) (formerly the National Center for Complementary and Alternative Medicine (NCCAM))	Government	Provides healthcare providers with information about several herbs and their potential interactions with other agents	Healthcare providers
Herbs at a Glance	Boddy et al. 2008 [52]; Jackson et al. 2001 [25]; Gregory et al. 2016 [42]; Motl et al. 2004 [28]	https://nccih.nih.gov/health/herbsataglance.htm	Factsheets/healthcare information	Website	Unclear	Yes	Yes	Yes, entirely free	National Center for Complementary and Integrative Health (NCCIH) (formerly the National Center for Complementary and Alternative Medicine (NCCAM))	Government	Provides basic information about specific herbs/botanicals, including common names, scientific evidence, potential side effects and cautions, and resources for more information	Healthcare providers, researchers, patients
KATIS (based off ALTMEDA)	Ogultarhan et al. 2016 [36]	http://www.komplementaeremedizin.de	Factsheets/healthcare information	Website	2016	Unclear	Unclear	Unclear	Authors	Researchers (authors)	A web-based information system for complementary medicine, designed to assist patients and health care professionals search for alternative therapies efficiently	Healthcare providers, patients
Medline Plus	Motl et al. 2004 [28]	https://medlineplus.gov/druginformation.html	Factsheets/healthcare information	Website	1998	Yes	Yes	Yes, entirely free	U.S. National Library of Medicine, National Institutes of Health	Government	Information resource on health, drugs and herbal supplements	Patients/public
Medscape	Motl et al. 2004 [28]; Spanakis et al. 2019 [46]	https://www.medscape.com/	Factsheets/healthcare information	Website and mobile app	1995	Yes	Yes	Yes, entirely free	WebMD (formerly SCP Communications Inc.)	Company	Provides clinical resources, news, and drug/disease information, as well as a drug interaction checker tool	Healthcare providers
PharmActa	Spanakis et al. 2019 [46]	No	Factsheets/healthcare information	Mobile app	Unclear	Unclear	Unclear	No	Authors	Researchers (authors)	An “app for personalized pharmaceutical care with information regarding drug-herbal medicinal products interactions” (Spanakis et al. 2019, p. 1 [46])	Patients
RxList (owned by WebMD)	Motl et al. 2004 [28]; Spanakis et al. 2019 [46]	https://www.rxlist.com/script/main/hp.asp	Factsheets/healthcare information	Website	1995	Yes	Yes	Yes, entirely free	WebMD (formerly a team of pharmacists)	Company	An online medical resource providing pharmaceutical information on brand and generic drugs and CAM, including an interaction checker and factsheets	Healthcare providers, patients/public
RxMed	Motl et al. 2004 [28]	https://www.rxmed.com	Factsheets/healthcare information	Website	1994	Yes	Yes	Yes, entirely free	RxMed Medical Advisory	Company	Provides illness and medication information, including CAM factsheets, as well as access to various medical products and services	Healthcare providers, patients
Search Engines (n = 4)												
Drugs.com	Motl et al. 2004 [28]; Spanakis et al. 2019 [46]; Yap et al. 2012 [39]	https://www.drugs.com/	Search engine	Website and mobile app	2001	Yes	Yes	Yes, entirely free	Cerner Multum and Thomson Micromedex (and Wolters Kluwer)	Company	Contains information about prescriptions and CAM therapies, as well as an interaction checker tool	Healthcare providers, patients/public
Electronic Medicines Compendium (EMC)	Motl et al. 2004 [28]	https://www.medicines.org.uk/emc/	Search engine	Website	1999	Yes	Yes	Yes, entirely free	Datapharm Ltd.	Company	Provides information, including leaflets, on drugs (including CAM) in the UK, as well as an adverse effect reporting tool	Healthcare providers (specifically pharmacists), patients/public
European Medicines Agency (EMA)	Spanakis et al. 2019 [46]	https://www.ema.europa.eu/en	Search engine	Website	Unclear	Yes	Yes	Yes, entirely free	European Medicines Agency	Government	Search tool to find information on herbal drugs and provides drug profiles	Healthcare providers, researchers, patients/public
Merck Manual	Motl et al. 2004 [28]	https://www.merckmanuals.com/en-ca/	Search engine	Website	1999	Yes	Yes	Yes, entirely free	Merck & Co. Inc.	Company	A search tool that allows users to find information on drugs and supplements (including side effects and interactions)	Healthcare providers, patients/public
Other (n = 6)												
Adverse Drug Reaction Information Bulletin (ADRIB)	Gao et al. 2015 [59]; Zhang 2018 [27]	http://www.sda.gov.cn/WS01/CL0078/ (inaccessible)	Bulletin	Website	2001	Unclear	Yes	Unclear	China Food and Drug Admininstration	Government	Technical bulletin that provides information on ADRs and potential hazardous drugs to highlight current pharmacovigilance concerns in China	Public
Cancer CAM™ Web-based continuing education program	Brink et al. 2004 [60]	http://www.nurse-ceus-stat.com/ (inaccessible)	Continuing education module	Web-based	2004	Unclear	Unclear	Unclear	HealthMark Multimedia	Company	To provide continuing education to nurses, allied health and health educators on the use of CAM for prostate cancer patients	Healthcare providers (including nurses and allied health professions), health/patient educators
Evaluation of Anthroposophical Medicine (EvaMed) Pharmacovigilance Network	Hamre et al. 2017 [61]; Tabali et al. 2012 [62]	No	Electronic Pharmacovigilance System	Unclear	2004	Unclear	Yes	No	Havelhoehe Research Institute	Researchers	Online adverse drug reaction reporting tool that connects with the hospital EMR system and integrates into the daily clinical practice of physicians	Healthcare providers, researchers
HIV Drug Interactions	Motl et al. 2004 [28]	https://www.hiv-druginteractions.org/checker	Interaction checker	Website	1999	Yes	Yes	Yes, entirely free	Liverpool HIV pharmacology group from the University of Liverpool	Researchers	Tool to check for interaction of HIV drugs and other drugs (includes complementary and alternative medicines)	Healthcare providers, researchers, patients
Ontology-based Artificial Neural Network Model for Drug Side-Effects	Yao et al. 2019 [63]	No	Model	Artificial Intelligence	2019	Unclear	Unclear	Unclear	Authors	Researchers (authors)	An ontology-based model for artificial intelligence-assisted medicine side-effect prediction	Healthcare providers, researchers
Herbopolis	Ee et al. 2018 [64]	https://play.google.com/store/apps/details?id=com.herbopolisgame.alpha&hl=en	Serious game	Mobile app	2017	Yes	Unclear	Yes, entirely free	Authors	Researchers (authors)	To motivate players to learn more about herbal medicine	Public

Table 3

**Table 3:** Frequency of updates by database identified via eligible articles

Database name	URL	Frequency of updates
ABDAMED	https://abdata.de/datenangebot/abdamed/	The content is updated daily.
Alticopeia Herbal Medicine Database	http://www.ddhsoftware.com/gallery.html?show=number&record=527	The website states that this database was last updated on January 11, 2009. The frequency of updates is not available.
ALTMEDA	Unclear	This database cannot be located outside the author’s original publication. The frequency of updates is not available.
American Botanical Council	http://abc.herbalgram.org/site/PageServer	Dates of updates to ABC’s product-specific and botanical ingredient monographs range from 2009 to 2019. Information on the frequency of updates made to ABC Clinical Guide to Herbs database is not available.
Caremark Drug Interactions	http://cpref.goldstandard.com/inter.asp?r=8084	Interaction reports list dates of revisions that range from 2016 to 2019. The frequency of updates is not available.
China Natural Products Database (CNPD)	Unclear	While this database has been mentioned in other papers, the database cannot be located, and the frequency of updates is not available.
Chinese Ethnic Minority Traditional Drug Database (CEMTDD)	http://www.cemtdd.com/	The database is still publicly available and maintained but no updates are apparent since the database was created 2014.
Clinical Pharmacology	https://www.clinicalpharmacology.com/	The content is updated daily.
Danish National Drug Interaction Database	http://www.interaktionsdatabasen.dk/	The website states that this database is updated “constantly”. The precise frequency of updates is not explicitly listed.
DoubleCheckMD	Unclear	While this database has been mentioned in other papers, the database cannot be located, and the frequency of updates is not available.
DrDrugs	https://www.skyscape.com/product/drdrugs-drug-guide-for-physicians	The exact frequency of updates will vary based on the publisher’s availability, but drug databases are updated on a monthly or quarterly basis.
Drug Information- formerly named DrugDigest	https://www.express-scripts.com/medco/consumer/ehealth/druginfo/dlmain.jsp?WC=N	The website states that the site homepage was last updated on 04/20/2020. However, dates are not listed for the drug profiles in the database, therefore the frequency of updates is not available.
Drug Product Database	https://www.canada.ca/en/health-canada/services/drugs-health-products/drug-products/drug-product-database.html	The website states that monographs are updated after a marketing drug has been authorized, but update frequency is not explicitly stated. Database appears to be updated up until 2020.
DrugBank	https://www.drugbank.ca/	The content is updated daily.
Encyclopedia of Traditional Chinese Medicine (ETCM)	http://www.nrc.ac.cn:9090/ETCM/	In their paper, Xu et al. 2019 [47] advised that the database would continue to be updated. The database is maintained but the frequency of updates is not explicitly listed.
Epocrates Rx (plus or pro)	https://online.epocrates.com/	The content is updated daily.
Guide to Popular Natural Products	http://www.skyscape.com/EStore/ProductDetail.aspx?ProductID=951 (inaccessible)	This database no longer exists. The frequency of updates when the database was in existence is not available.
IBIS: Integrative BodyMind Information System	http://www.ouribis.com/	Update frequency is not mentioned clearly. The database was not working for a period of time, and became available again in 2010, The last update appears to be in 2013.
International Drug Information System (INTDIS):	N/A	The INTDIS no longer exists, as it has been replaced by Vigibase.
Lexi-Natural (includes LexiComp)	http://webstore.lexi.com/Store/Individual-Databases/Lexi-Natural-Products	The content of LexiComp is updated daily. It is stated that the online version has been updated 1–5 time(s) a year, but the last update is recorded as June 2018.
MedicinesComplete (includes Herbal Medicines; formerly known as the British National Formulary)	https://about.medicinescomplete.com/publication/herbal-medicines/	MedicinesComplete is updated 3–4 times a year, usually in January, April, July, and October.
Micromedex (includes DrugDex, Drug-Reax, AltMedDex)	https://www.micromedexsolutions.com/home/dispatch/ssl/true	The content is updated daily.
Natural Medicines (formerly Natural Medicine Comprehensive Database (NMCD) and Natural Standard Database (NSD))	https://naturalmedicines.therapeuticresearch.com/	The content is updated daily.
OncoRx Database (called Onco-Rx as a website, and OncoRx-MI as a mobile app)	http://www.onco-informatics.com/oncorx/	The update frequency is not clear, though likely at least annually. The last update was reported in 2019.
PEPID Drug Information Database	https://www.pepid.com/	Update frequency is not mentioned clearly. It was mentioned that the content is updated “continuously” and “regularly”.
PharmDB-K	http://biomart.i-pharm.org http://pharmdb-k.org/	There is no information available. Neither links provided lead to an accessible website.
PharmGKB	https://www.pharmgkb.org	Literature annotations and variant annotations are added in real time as a curator annotates the paper. Very important pharmacogene (VIP) summaries, curated pathways and clinical annotations are reviewed periodically. New clinical guideline annotations and drug label annotations are added as they become evident. Gene, drug and disease data that is automatically retrieved is updated in response to scheduled updates at the external resource.
Tarascon Pocket Pharmacopoeia	https://www.tarascon.com/products/mobile/	The frequency of updates is not clear, but the last update was in 2020.
TCM Assistant	http://www.tcmassistant.com/	There is no information available regarding update frequency, and the website is inaccessible.
TCM Database@Taiwan	http://tcm.cmu.edu.tw/	There is no information available regarding update frequency, and the website is inaccessible.
TCMGeneDIT	http://tcm.lifescience.ntu.edu.tw/	There is no information available regarding update frequency, and the website is inaccessible.
TCM-MESH	http://mesh.tcm.microbioinformatics.org/	There is no information available regarding update frequency, and the website is inaccessible.
Traditional Chinese Medicine Information Database (TCM-ID)	http://bidd.nus.edu.sg/group/TCMsite/ New URL: http://119.3.41.228:8000/tcmid/ 2017 Version: Huang L, Xie D, Yu Y, Liu H, Shi Y, Shi T, Wen C. TCMID 2.0: a comprehensive resource for TCM. Nucleic acids research. 2018 Jan 4;46(D1):D1117–20.	The first version of TCM-ID was released in 2012. Since this time, however, a second version was released in 2017 (see new URL with accompanying citation). The frequency of updates since 2017 is unclear.
Vigibase (includes Vigilyze)	https://www.who-umc.org/vigibase/vigibase/	VigiBase is continuously updated with reports, sometimes daily, as they are submitted by member countries of the World Health Organization Programme for International Drug Monitoring. Most national centres report quarterly or even more frequently. The date of occurrence is noted when supplied, and the date of entry into VigiBase is recorded.

Table 4

**Table 4:** Databases created and/or updated within the last 3 years identified via eligible articles

Databases	URL	Summary
**Publicly Available**		
American Botanical Council	http://abc.herbalgram.org/site/PageServer	The American Botanical Council provides a searchable database titled “ABC Clinical Guide to Herbs” that includes monographs, clinical overviews, and patient information sheets for various herbs. While access to this database requires a paid subscription, the resource also offers free databases with information on herbal and plant-based ingredients, herb profiles and select herbal monographs.
Caremark Drug Interactions	http://cpref.goldstandard.com/inter.asp?r=8084	Copyrighted through Gold Standard, the Caremark Drug Interactions database includes a drug-interaction search tool. Reports are provided for interactions between drugs, CAM, food, alcohol, and other substances.
Danish National Drug Interaction Database	http://www.interaktionsdatabasen.dk/	The Danish Medicines Agency provides a searchable interactive database for prescription drugs, herbal remedies, vitamins, minerals, and grapefruit juice.
Drug Information- formerly named DrugDigest	https://www.express-scripts.com/medco/consumer/ehealth/druginfo/dlmain.jsp?WC=N	The Drug Information database includes a search tool to find monographs and information on medications, herbal drugs, and supplements. While several drugs are indexed in the database, the majority of drugs do not have a monograph available.
Drug Product Database	https://www.canada.ca/en/health-canada/services/drugs-health-products/drug-products/drug-product-database.html	The Drug Product Database is maintained by Health Canada through the Government of Canada and contains a searchable database for drugs authorized for sale in Canada. While several drugs are indexed in the database, many do not have product monographs available.
DrugBank	https://www.drugbank.ca/	The Drugbank database provides information on drugs and drug/chemical target information.
Encyclopedia of Traditional Chinese Medicine (ETCM)	http://www.nrc.ac.cn:9090/ETCM/	ETCM is an online searchable database that provides data on the ingredients, herbs, and formulas of Traditional Chinese Medicine.
PharmGKB	https://www.pharmgkb.org	PharmGKB is a searchable pharmacogenomic database targeted for researchers that provides data on the relationship between genetic variations and drug response. Molecules, genes, or variants can be searched within the database.
Traditional Chinese Medicine Information Database (TCM-ID)	http://119.3.41.228:8000/tcmid/	In its second revision, the TCM-ID database provides information on the relationships between the herbs and formulas and the pharmacology or biochemistry of Traditional Chinese Medicine.
**Requiring Paid Subscription**		
Clinical Pharmacology	https://www.clinicalpharmacology.com/	Clinical Pharmacology is a drug information resource that is powered by Clinical Key and owned by Gold Standard. The database contains monographs for prescription drugs as well as CAM products.
DrDrugs	https://www.skyscape.com/product/drdrugs-drug-guide-for-physicians	DrDrugs provides a mobile and web-based database of monographs for drugs and herbal products.
Epocrates Rx (plus or pro)	https://online.epocrates.com/	Epocrates offers a web-based and mobile version of their database of drug monographs and interaction reports.
Lexi-Natural (includes LexiComp)	http://webstore.lexi.com/Store/Individual-Databases/Lexi-Natural-Products	Lexi-Natural is part of the Lexicomp database and includes drug monographs and patient handouts for both prescription drugs and CAM products.
MedicinesComplete (includes Herbal Medicines; formerly known as the British National Formulary)	https://about.medicinescomplete.com/publication/herbal-medicines/	Developed by the Royal Pharmaceutical Society, MedicinesComplete provides a database of drug and CAM monographs and drug interaction reports.
Micromedex (includes DrugDex, Drug-Reax, AltMedDex)	https://www.micromedexsolutions.com/home/dispatch/ssl/true	IBM Micromedex provides a searchable database of drug and CAM monographs as well as disease and toxicology
Natural Medicines (formerly Natural Medicine Comprehensive Database (NMCD) and Natural Standard Database (NSD))	https://naturalmedicines.therapeuticresearch.com/	Natural Medicines is a database with several sub-databases on databases on food, herbs and supplements, herbal combinations, drug manufacturers, sports medicine, health and wellness and a comparative effectiveness database. The database also features tools including an interaction checker, effectiveness checker, nutrient depletion, pregnancy and lactation checker and adverse event checker.
OncoRx Database (called Onco-Rx as a website, and OncoRx-MI as a mobile app)	http://www.onco-informatics.com/oncorx/	Onco Rx is a database of interaction information for oncology drugs, chemotherapy regimens and CAM products.
PEPID Drug Information Database	https://www.pepid.com/	PEPID Knowledgebase is a web-accessible database hosted through PEPID Connect. Features of PEPID Connect include: Drug interaction checker; drug-allergy checker; pill identification; IV Compatibility tool; lab manuals; clinical calculators; drug database; differential diagnosis and symptom checker; news and alerts; PEPID PGx pharmacogenomic tool.
Tarascon Pocket Pharmacopoeia	https://www.tarascon.com/products/mobile/	The Tarascon Pocket Pharmacopoeia database contains drug guides and information on drug interactions. The database comes in a PDA format and also includes information on herbal supplements.

## Abbreviations

CADTH: Canadian Agency for Drugs and Technologies in Health
CAM: Complementary and alternative medicine
CAHTS: Computer-assisted history-taking systems
CDSS: Computerized decision support systems
mHealth: Mobile health
NCCIH: National Center for Complementary and Integrative Health
NHP: Natural health product
